# Mobile phone data analyses for public health research: a scoping review

**DOI:** 10.3389/fpubh.2025.1728985

**Published:** 2025-11-20

**Authors:** Xuening Cheng, Wei Jiang, Siyi Liu, Xuyan Lou, Yang Li, Lefan Liu, Molin Li, Xuyang Wang, Yan Cen, Alain Chong, Zhuo Chen

**Affiliations:** 1 School of Economics, Faculty of Humanities and Social Sciences, University of Nottingham, Ningbo, China; 2 College of Accounting, Ningbo University of Finance and Economics, Ningbo, China; 3 Kelley School of Business, Indiana University, Bloomington, IN, United States; 4 Department of Applied Health Sciences, University of Birmingham, Birmingham, United Kingdom; 5 Smartsteps Data Technology Co., Ltd., Beijing, China; 6 Nottingham University Business School, University of Nottingham, Ningbo, China; 7 Department of Health Policy and Management, College of Public Health, University of Georgia, Athens, GA, United States

**Keywords:** mobile phone data, public health, mobile signaling data, population mobility, pandemic

## Abstract

Mobile phone data provide high-resolution, near real-time measurements of population mobility and have become an increasingly valuable source for public health research, enabling rapid evaluation of policy impacts on human movement and pandemic control. However, the methodological challenges surrounding the extraction, governance, and validation of mobile phone data for the public health community remain insufficiently explored. Following the PRISMA-ScR framework, we conduct a scoping review to synthesize major research themes, opportunities, and challenges in the use of mobile phone data for public health, particularly pandemic-related studies. Our findings highlight limitations in the empirical use of these datasets, including demographic and population coverage, representativeness, and equity issues, as well as the transparency of data extraction and processing. We also provide guidance for future research, including the development of standardized frameworks for data curation and validation, a clear understanding of algorithms that extract mobility information, and rigorous interpretation of mobility metrics.

## Introduction

Human mobility, the movement of human beings across space and time, is a fundamental determinant of disease transmission and public health dynamics (1, 2). However, measuring and monitoring human mobility has traditionally been challenging due to limited data availability and granularity (3). The widespread use of mobile phones and advances in big data technologies have transformed this landscape, enabling large-scale, near-real-time tracking of human mobility (4), offering insights that were previously unattainable (5). Capturing location information at high spatial and temporal resolutions (6), mobile phone data allows continuous, rapid, and detailed analysis of human behavior far beyond the capacity of traditional survey data (3).

In recent years, mobile phone data have attracted significant attention within the public health community, as they provide valuable information for assessing the impact of health policies and understanding transmission dynamics of infectious diseases (4, 7). They support surveillance, forecasting, and evaluation of pandemics and have been widely used to analyze the effects of Non-Pharmaceutical Interventions (NPIs) on population mobility (8). Diverse analytical perspectives and models have been developed to study human behavior based on spatiotemporal features extracted from various types of mobile phone data, facilitating research on mobility patterns, social networks, disease transmission, and the evaluation of control strategies (5). Despite these advances in mobile phone data, concerns regarding personal privacy, data governance, and methodological challenges persist, alongside the potential for misuse of the data (5, 9).

Mobile phone generated data can be broadly categorized into two types based on the source and the method of collection: signaling data (and call detail records, CDRs) generated by phones during communication with cell towers, and GPS/App-based data generated by mobile Apps (Table 1). Mobile phones frequently ping nearby cell towers, leaving a record of the signal data that can be used to triangulate the phone’s location based on antenna connections (10). A key advantage of mobile phone signaling data is that it provides location information as long as the mobile phone is switched on, even if it is not actively being used. CDRs typically contain the user ID, timestamp, and cell tower location associated with calls, texts, or other activities tied to the SIM card (7). In another way, GPS/App-based data generated by Apps such as Google Maps and Baidu Maps, often use global positioning system (GPS) services to record a device’s location over time. GPS/App-based data offer greater accuracy in terms of spatial precision and provide more frequent observations than mobile signaling data (7). However, these data are usually aggregated for privacy protection, often presented as population-level data at specific point-of-interest (POIs) or locations. While GPS/App-based data provide finer location details than mobile phone signaling data, the latter is more representative of the broader population since it is not limited to users of specific applications.

**Table 1:** Comparing different types of mobile phone generated data.

Type	Subtype	Description	Providers	Pros	Cons
Mobile phone data	Mobile phone signaling data	Continuous location information about the cell tower that a handset is connected to as long as it is switched on	Mobile service operators	Substantial coverage of population Passive continuous connectivity logs without user consent permission	Limited spatial and temporal granularity of data Duplicate identification in multi-SIM user scenarios
	Call detail records (CDRs)	Duration, timestamp and location information of user communication activities	Mobile service operators	Cost-effectiveness and high availability With population representativeness	SIM card event-driven records with discontinuity Duplicate identification issues for multi-SIM users Limited spatiotemporal resolution
GPS/App-based data	GPS related data from location intelligence firms	GPS data collected and aggregated from smartphone applications	Major Tech firms (such as Google, Twitter, Baidu)	Precise location information	Lack of transparency in data generation Lack of representativeness Restricted outputs indexes

Despite the growing interest in and utilization of mobile phone data in public health, significant methodological challenges are associated with data extraction, data governance, and data quality for mobile phone signaling data (3, 6). While there has been extensive research using mobile phone data, no comprehensive review has focused specifically on the empirical use of mobile phone signaling data in public health. To address this gap, this article conducts a scoping review on the use of mobile phone signaling data in public health. Our review focuses on the mobile phone signaling data, while the GPS/App-based mobile phone data warrants a separate review. We have curated an extensive list of publications on the use of mobile phone data in public health, synthesizing key themes, highlighting both the opportunities and challenges of its application in pandemic response efforts, and suggesting avenues for future research.

## Methods

### Overview

The scoping review follows the PRISMA-ScR (Preferred Reporting Items for Systematic Reviews and Meta-Analyses Extension for Scoping Reviews) guidelines and adopts the Arksey and O’Malley methodology framework (11).

### Identifying the research question

The primary aim of this review was to examine the classification, application, and methodological, societal, and data-related challenges and opportunities of the empirical use of mobile phone data in public health. The core research questions are:

(1) How have mobile phone data been used in public health research, specifically for pandemic control?
(2) What are the key opportunities and challenges associated with the use of mobile phone data for public health research?

### Identifying relevant studies

The search strategy was developed by WJ, XC, and ZC. We conducted a search across four major citation databases: MEDLINE, PubMed, ScienceDirect, and Web of Science, covering publications in English from January 1, 2012, to June 30, 2024. The search strategy combined keywords related to mobile phone data (“Cell phone” OR “Mobile Phone”) and mobility terms (“Mobility” OR “Flow”) along with the pandemic (“COVID” OR “Pandemic”). The search strategy was refined to ensure comprehensive coverage of relevant themes (see Appendix Table 1 for details).

### Study selection

Inclusion criteria of the review were: (1) studies focused on population mobility, human movement, or related patterns; (2) studies clearly described the applications of mobile phone data for illustrating or measuring mobility patterns; (3) studies provided empirical evidence in the context of the pandemic. Two reviewers (XC and WJ) independently conducted the blind review process, screening titles and abstracts to exclude studies that did not pertain to mobility, pandemics, or healthcare utilization, secondary studies (e.g., review articles), theoretical studies without empirical applications, and studies lacking details on data sources. The full texts were assessed by the two reviewers based on the metrics of the mobile phone data and the study outcomes. Discrepancies when there was uncertainty (44%^†^) or inconsistent decisions (9%^†^) were resolved by a senior reviewer (ZC).

### Charting the data

Data from the included studies were charted by two reviewers (XC and WJ), who identified key characteristics, including study title, research theme, study region, study population, data source, mobility metrics, main outcomes, and study findings. According to scoping review guidelines, no studies were excluded based on quality, as the aim was to identify gaps in the literature rather than assess methodological rigor (11).

### Collating, summarizing, and reporting the findings

**Figure 1:**
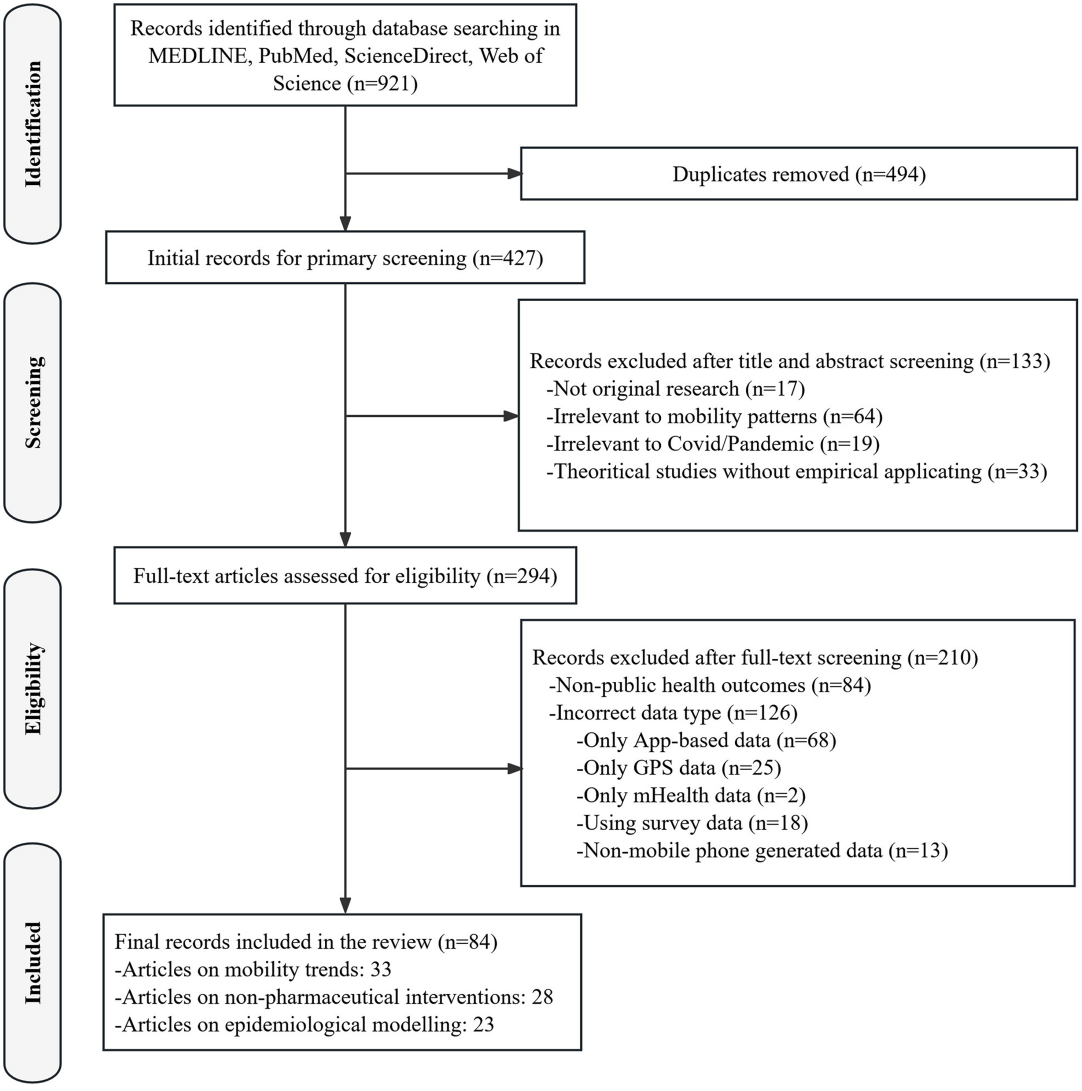
PRISMA flowchart of the literature search process.

A PRISMA flowchart (Figure 1) was developed to track the selection process. The findings were synthesized to identify key patterns and relationships across studies, and a Sankey diagram was used to visualize the distribution of research themes, mobility metrics, and outcome domains.

## Results

### Overview

A total of 921 records were retrieved from database searches and imported into a local citation database via Zotero 6.0. After de-duplication, it yielded a list of 427 citations. Following an initial screening of titles and abstracts, 133 citations were excluded due to irrelevance. Among the remaining 294 full-text articles, 210 were excluded because of non-public health outcomes (*n* = 84) or incorrect data type (*n* = 126; specific reasons for exclusion are presented in Figure 1). Ultimately, 84 articles met the inclusion criteria and were included in the final review. A narrative and numerical summary of study characteristics was presented and tabulated in the Appendix Table 2. Four key themes with important implications emerged from our review of mobile phone data applications in pandemic control-related research.

### Tracking population mobility

A total of 25 studies (30%) focused on analyzing mobility patterns using mobile phone data. Jia and colleagues were the first to use real-time mobile phone data to assess the spatio-temporal dynamics of the COVID-19 spread in China (12). Using national aggregated mobile operator data, Lu et al. established the correlation between population mobility and disease transmission (1). Luo et al. analyzed the mobility patterns among different populations and how they influence the risks of disease transmission (13). Aside from China, an extensive list of research used mobile phone signaling data in public health research exists, analyzing the associations between human mobility and coronavirus spread in the US (14–19), Latin America (20), Ireland (4), Spain (6, 21), Brazil (22), Ecuador (23), Japan (24), Finland (25), and globally (26). However, in the later stages of the pandemic, the association between mobility metrics derived from mobile phone data and COVID-19 incidence growth rates gradually weakened following the lifting of initial stay-at-home orders (27). Other studies explored the socioeconomic impacts of mobility changes, linking mobility data with demographic characteristics (21, 28), economic distress (16), and health outcome indicators (29).

### Evaluation of non-pharmaceutical interventions

Thirty-six studies (43%) evaluated the effectiveness of multiple NPIs using mobile phone data, which were adapted as primary measures to restrain the spread of COVID-19 globally before the introduction of effective vaccinations. Many studies assessed social distancing (30–39) and lockdown policies (40–46) across different countries, highlighting their role in mitigating the spread of COVID-19. Research also showed that less aggressive interventions, such as remote working and closures of non-essential businesses, had varied impacts depending on the country (47–54). Notably, a study covering 135 countries found significant reductions in mobility due to travel restrictions during the first wave of the pandemic (55); however, other studies emphasized spontaneous reductions in mobility that occurred regardless of government actions and a ‘floor’ phenomenon (19).

### Predictive modeling of disease transmission

Incorporating mobility parameters—either tuned or estimated from mobile phone data—into epidemiological models enhances their ability to simulate outbreak dynamics under varying conditions. A total of 23 studies (27%) integrated mobile phone data into epidemiological models to predict disease spread. These models helped simulate outbreak trajectories in various regions, including Chinese cities and worldwide (56–69). A notable study combined mobile phone data with genomics to track coronavirus variants in Bangladesh, revealing how large-scale human migration from urban to rural areas influenced viral diversity (70). Social factors, such as sociodemographic characteristics, were also examined, showing how mobility differences contributed to infection rates in disadvantaged groups (56, 71). Other researchers highlighted that socioeconomic factors, such as education, household size, and the proportion of the Latinx population, have consistent positive relationships with COVID-19 prevalence over time (72).

### Implications for health equity

A sheer body of studies highlighted the potential of mobile phone data to analyze health inequality issues. Research on gender-specific mobility patterns (73), age-group mobility (74–76), and socioeconomic status (SES) (77–79) revealed disparities in COVID-19 transmission risks. Studies also showed that NPIs affected populations and communities differently based on SES, influencing the mobility responses and, consequently, infection rates (44, 80, 81). For instance, using mobility measures derived from mobile phone data, Carranza et al. showed that the impact of NPIs on mobility varied widely across communities, depending on their socioeconomic levels, which also contributes to disparities in infection rates between high- and low-income areas (53).

## Discussion

Our scoping review highlights the emerging role of mobile phone signaling data as a valuable alternative for population mobility analysis in public health. The aggregation and analysis of such large-scale, routinely generated data represent a major advancement in digital epidemiology (38). Mobile phone data have increasingly been adopted in modeling population flows, simulating the spatiotemporal transmission dynamics, thereby enhancing the accuracy of risk assessments and informing public health response strategies. More importantly, our review underscores several key considerations and challenges that must be aware to fully realize the potential of mobile phone data in public health.

### Measurement of mobility

**Figure 2:**
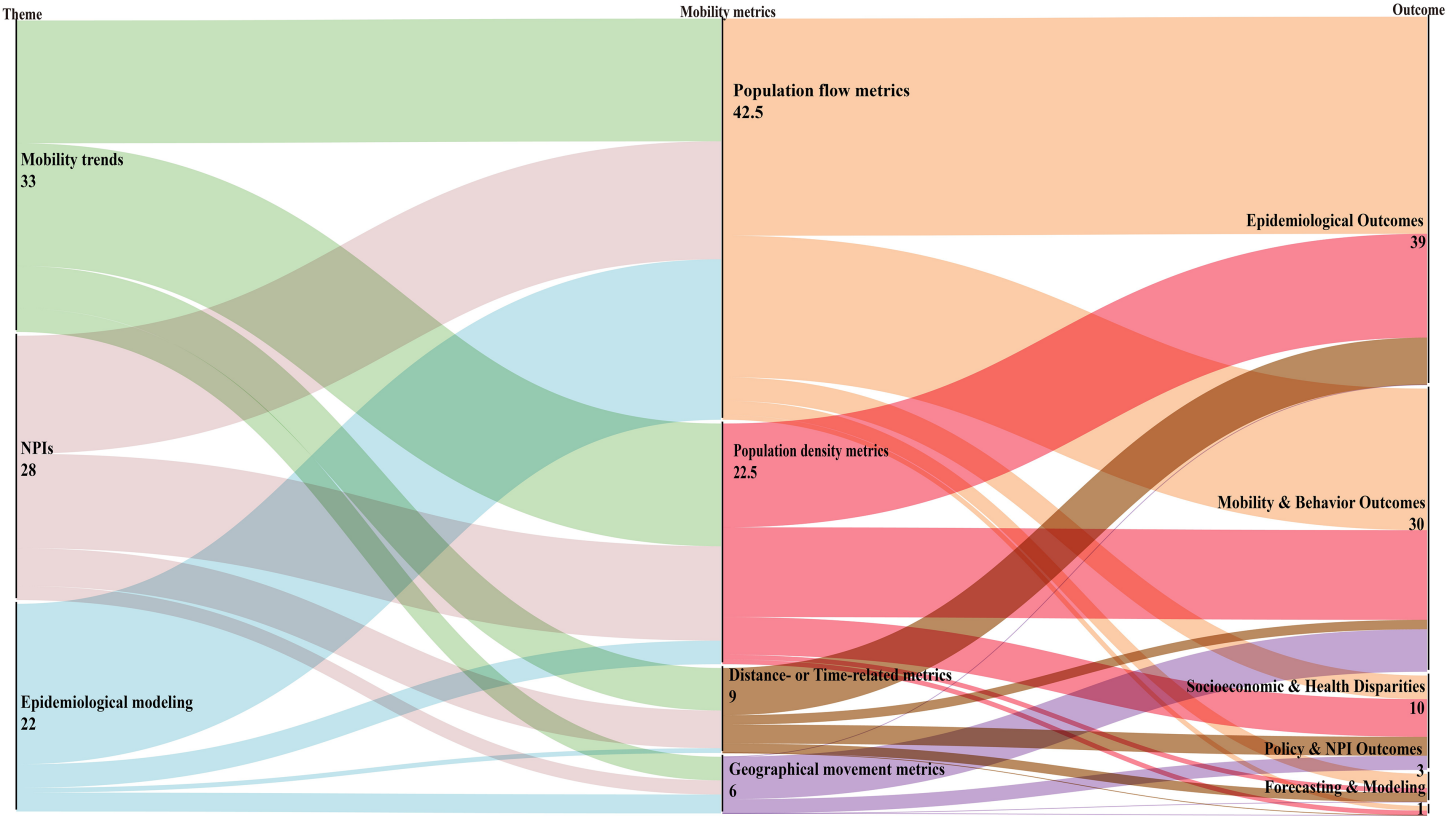
Sankey diagram illustrating the distribution of included studies from research themes (left) through mobility metric classes (middle) to main outcome domains (right). Link widths are proportional to the number of studies. We categorized mobility metrics into four non-exclusive classes: Population flow, Population density, Geographical movement, and Distance/Time. Because individual studies often used multiple classes, we applied fractional counting: if a study used k classes, each Theme-to-Metric link received a weight of 1/k. Duplicates within the same class were counted once per study. For transparency, a full-counting version of metric co-usage is provided in Appendix Table 2.

Mobile phone data enable the measurement of human mobility through diverse metrics, supporting multidisciplinary research. As illustrated in the Sankey diagram (Figure 2), we summarize mobility metrics into four major categories. Population flow metrics, such as origin–destination flow indicators, can track the movement of infected individuals across regions, revealing transmission pathways (1, 6, 62). Population density metrics, including the number of visits or hourly population density to POIs, help evaluate the implementation and effectiveness of pandemic control policies across different population groups (56, 82). Geographical movement indicators, such as the radius of gyration or entropy of movement, can be used to refine epidemiological models and simulate disease transmission (9, 59). Time- or distance-related metrics—such as duration spent at specific locations, distance traveled, travel frequency, and activity space—capture behavioral response to public health interventions (31, 40, 48). Additional information is presented in Appendix Table 3.

However, the diversity of mobility metrics also introduces challenges. Comparisons between studies become complicated, and the predictive power of different mobility indicators in empirical models varied substantially across regions and time period (15, 83). The reliability of mobility metrics derived from mobile phone data as proxies for disease transmission is inconsistent, temporally and geographically. For instance, the correlation between alternative mobility metrics and the effective reproductive number of the coronavirus fluctuated during the early stages of COVID-19 (59). These findings underscore the importance for researchers of carefully selecting appropriate mobility indicators and ensuring their robustness when interpreting results or informing health policymaking.

### Lack of transparency in data generation

The procedures and algorithms used to collect and process mobile phone raw data are often opaque to public health and social science researchers. Many studies do not provide a detailed description of how the raw data is generated. For example, the frequency of mobile signal pings used to define a device’s location (e.g., every 5 min versus every 10 min) can significantly impact mobility estimates and the inferred effect of interventions (59). Data generation is also influenced by factors such as cell tower distribution and the types of mobile applications used, which can vary from urban to rural areas and across countries (35). This complexity and lack of transparency in data generation hinder the ability to link mobility data to human behavior (59), which may reduce the reliability and trustworthiness of subsequent analyses.

### Data privacy

As the large-scale use of mobile phone data becomes more common, protecting individual privacy remains a central concern (84). To minimize privacy risks, data providers often aggregate and preprocess data through methods such as statistical thresholds, differential privacy, and appropriate security controls (35), providing researchers with spatially and temporally aggregated datasets. While these approaches safeguard privacy, they reduce data granularity and can introduce uncertainties in data quality (7), which may limit the comparability and generalizability of results across studies.

### Representativeness and equity implications

Mobile phone data enable rapid, large-scale, and context-specific data collection (85), but representativeness requires careful consideration. Representativeness of mobile phone data depends on market share, user demographics, and geographic coverage. Data from a single operator or regions with uneven network coverage may systematically underrepresent children, older adults, and rural or low-income populations, limiting the generalizability of results and potentially biasing conclusions about behavioral responses to NPIs (84–86). During the COVID-19 pandemic, mobility reductions were found to be smaller in low-income communities due to occupational and structural constraints, suggesting that operator coverage biases may distort inferences on health equity and assessments of policy effectiveness (53, 80). To mitigate these issues, recent studies have recommended corrective strategies such as reweighting using census covariates, small-area estimation to improve spatial representativeness, and integrating multiple mobility and demographic data sources (14, 84, 85, 87). Strengthening such representativeness adjustments is crucial to ensure that mobility-derived evidence makes a meaningful contribution to equity-oriented public health research.

### Time and spatial scales for aggregation

Selecting the appropriate spatial and temporal scales is critical for striking a balance between privacy protection and analytical utility. Spatial units may include grids or an administrative area, which should reflect population density differences between urban and rural regions. Temporal scales should align with study designs and research objectives; for example, daily measures can better indicate short-term disease transmission, whereas weekly measures can better reflect seasonal or migration patterns (35). Flexible aggregation ensures mobility metrics are informative, actionable, and ethically responsible.

## Future research directions

Our review shows that mobile phone data have been extensively used to measure population mobility, model disease transmission, and guide and assess public health interventions for pandemic control. These data offer near-real-time insights into large-scale population behavior, and hold promise for both retrospective and prospective public health analyses (88). However, practical challenges highlighted in our review and previous study (84) underscore the need for cautious and informed application. The following directions may help better harness these data for public health outcomes of interest, enabling a more effective understanding of population behaviors and responses in rapidly changing situations.

### Understanding the data generation process

A major challenge is the limited understanding of how these data are collected, processed, and transformed into usable metrics. Current preprocessing methods for mobile phone mobility data often operate as black boxes for researchers, which may lead to biases in analyses with ambiguous directions (84). Researchers must critically examine the assumptions underlying default conditions and thoroughly understand the critical decision rules (35), such as the spatial boundaries of POI, the minimum interactions required to register activity, and the criteria defining “stay” or “pass-by” actions. Transparent reporting of these technical details is crucial for replicating, validating, and scaling findings across academia, industry, and policy contexts. A list of recommended reporting items is in Appendix Table 4.

### Exploring more granular data

Current uses of mobile phone datasets are typically anonymized and aggregated to preserve privacy. Although useful for broad mobility trends, such aggregates may be insufficient for targeted public health responses, especially in the post-pandemic era, where sustained surveillance is required (84, 85). In addition, there is a growing need to access finer, sub-population-level mobility data to capture the heterogeneous transmission patterns and disproportionate epidemic burdens, as our review identified. Therefore, there is a need to bolster open discussions and collaborations among mobile operators, policymakers, and researchers to develop frameworks that ensure privacy while enabling legally compliant extraction of detailed, actionable mobility information.

### Linkage with other data sources

A new and growing body of literature explores the integration of mobile phone data with complementary datasets, such as census demographics (20, 21, 79), social media (33, 82), internet search volume (16), or genomics (70). With its extensive coverage and spatiotemporal scale, integrating mobile phone data with other data sources can overcome user-based selection bias, provide a more comprehensive picture of population activity (84), and enhance understanding of the profound social and health equity implications. Linking mobility data with socioeconomic and behavioral information at an appropriate geographic scale (e.g., census block group level) enables a more comprehensive view of transmission patterns, disparities, and intervention effectiveness (7), supporting more effective and informed public health decision-making.

## Limitations

This review has three main limitations. First, it focuses primarily on the use of mobile phone data for pandemic control, potentially overlooking applications in other public health areas such as influenza or tuberculosis control. Second, the review is limited to English-language health literature, excluding studies published in languages other than English or in engineering and technical fields. Third, mobile phone data also has significant analytical value in other research fields, such as urban planning, emergency evacuation, and economic resilience and forecasting (84), while our review concentrates on public health applications.

## Conclusion

Mobile phone data have become an increasingly valuable tool for studying human mobility across multidisciplinary research and practices, as evidenced by the expanding body of literature referring to this data. Our review shows that these data are primarily used to characterize spatial and temporal mobility patterns, assess the role of human movement in disease spread, and perform simulation and predictive modeling of outbreaks. Despite their substantial potential, as we reviewed, challenges remain in ensuring inclusive, transparent, and sustainable use (9). Critical information on user demographics, population coverage, device usage differences, and data processing remains incomplete (59), complicating comparisons across studies and settings. Future work should prioritize validation, standardized frameworks for data curation, and transparent reporting of processing algorithms to strengthen the interpretability and reliability of mobility metrics in public health research.
