# Supplementary material for: Mobile phone data analyses for public health research: a scoping review
Source: Front Public Health. 2025 Nov 20;13:1728985. doi: 10.3389/fpubh.2025.1728985 (PMC12675487; doi:10.3389/fpubh.2025.1728985)
Supplement: Supplementary file 1 [file Table_1.docx]

***Supplementary Documents***

**Mobile Phone Data Analyses for Public Health Research: A Scoping Review**

Appendix Table 1: Searching Strategies Used for the Scoping Review

| **Database** | **Searching Strategy** |
| --- | --- |
| MEDLINE | 1. (("Cell phone"[Title/Abstract] OR "Mobile Phone"[Title/Abstract]) AND ("Mobility"[Title/Abstract] OR "Flow"[Title/Abstract]) AND ("Covid"[Title/Abstract] OR "Pandemic"[Title/Abstract]))  2. (("2012/01/01"[Date - Publication]: "2024/06/30"[Date - Publication]))  3. 1 AND 2 |
| PubMed | 1. (("Cell phone"[Title/Abstract] OR "Mobile Phone"[Title/Abstract]) AND ("Mobility"[Title/Abstract] OR "Flow"[Title/Abstract]) AND ("Covid"[Title/Abstract] OR "Pandemic"[Title/Abstract]))  2. (("2012/01/01"[Date - Publication]: "2024/06/30"[Date - Publication]))  3. 1 AND 2 |
| ScienceDirect | 1. Title, abstract or author-specified keywords: (“Cell phone” OR “Mobile Phone”) and (“Mobility” OR “Flow”) and (“Covid” OR “Pandemic”)  2. Published date: 2012-01-01 to 2024-06-30  3. 1 AND 2 |
| Web of Science | 1. Topic (title, abstract, or keywords) : (“Cell phone” OR “Mobile Phone”) and (“Mobility” OR “Flow”) and (“Covid” OR “Pandemic”)  2. Published date: 2012-01-01 to 2024-06-30  3. 1 AND 2 |
