# Supplementary material for: Mobile phone data analyses for public health research: a scoping review
Source: Front Public Health. 2025 Nov 20;13:1728985. doi: 10.3389/fpubh.2025.1728985 (PMC12675487; doi:10.3389/fpubh.2025.1728985)
Supplement: Supplementary file 4 [file Table_4.docx]

| Appendix Table 4: Recommended Reporting Items for Mobile Phone Data Analysis | |
| --- | --- |
| Items | Definition (for Mobile Phone Data Analysis) |
| *Strongly Recommended* | |
| Sampling Cadence | The frequency at which a mobile device's location is recorded or "pinged." In CDR data, this is passive (e.g., only when a call/SMS occurs) or at a time interval (e.g., every 5 minutes or every 1-2 hours). |
| Ping Logic | The underlying mechanism or rules that determine when and why a device's location is recorded and transmitted. This may be time-based, distance-based, speed-based, or triggered by specific network events (like connecting to a new cell tower). |
| Stay-Point Rules | Algorithms used to identify meaningful stops (e.g., home, work, shopping) in a user's trajectory data, filtering out movement noise (like GPS drift). They typically involve thresholds for minimum time spent in a specific area and a maximum distance radius. |
| Aggregation Thresholds | The minimum size or count (e.g., number of devices, minimum time period, minimum geographic area) required before data can be published or analyzed. This is crucial for maintaining data privacy and preventing the re-identification of individual users. |
| Privacy Noise | A privacy-preserving technique where random uncertainty (noise) is intentionally introduced to the data (e.g., in location coordinates or counts) to protect individual identities while still allowing for aggregate analysis of the population. |
| *Recommended if Available* | |
| Tower Density | The concentration of cell towers in a given geographic area. Higher density (e.g., in a city) means more accurate location estimates, as a device is closer to a tower; lower density (e.g., in a rural area) means less precise location estimates. However, this information may not be available or location-specific. |
| Device Coverage by Age/SES/Region | The extent to which the analyzed mobile data accurately represents the true demographic structure of the population, specifically how well devices are distributed across age groups, socioeconomic status (SES), and geographic regions. |
| Validation References | External, reliable data sources (e.g., official census data, transportation surveys, traffic counts, government statistics) used to check and correct potential biases and errors in the mobile phone data analysis, ensuring the results are generalizable and accurate. |
